# Training an infectious disease unit in palliative care during and post COVID-19: a qualitative longitudinal study

**DOI:** 10.3389/fpubh.2024.1393770

**Published:** 2024-10-16

**Authors:** Sara Alquati, Giovanna Artioli, Gianfranco Martucci, Silvia Tanzi

**Affiliations:** ^1^Palliative Care Unit, Azienda USL – IRCCS di Reggio Emilia, Reggio Emilia, Italy; ^2^Department of Medicine and Surgery, University of Parma, Parma, Italy; ^3^Local Health Unit of Modena, Modena, Italy

**Keywords:** COVID-19, palliative care, qualitative research, education, infectious disease

## Abstract

**Background:**

To understand palliative care needs and their changes perceived by health professionals (HPs) of the Infectious Diseases Unit who participated in palliative care (PC) intensive training during the pandemic and behind/during the pandemic and one year after the outbreak.

**Methods:**

A longitudinal qualitative study. Thematic analysis and meaning shift were two months after training to one year. This specific thematic approach enabled the researchers to fully understand the experiences of the HPs after they participated in the intensive PC training program during the pandemic. Participant validation meeting with the ward’s staff one year after the end of the course was performed. The two last validation meetings were used as a triangulation source to plan the new education projects.

**Results:**

From March 9 to 28, 2020, the Palliative Care Services (PCS) developed intensive experiential training. Thirty-one HPs of the Infectious Diseases Unit (physicians and nurses) who were facing the COVID-19 emergency participated in the training. We conducted eight semi-structured interviews with HPs who participated in intensive training during the first wave of the pandemic (T0), two months (T1) after training and after one year (T2), during the second wave. Two validation meetings were performed as suggested by the best practices in medical education. Twenty-two infectious disease staff members participated, 8 physicians and 14 nurses. Our data show a meaning shift on five overarching themes (defined within the sub-themes): (1) Recognizing patients’ palliative care needs; (2) Responses to palliative care needs; (3) Increasing attention to intervention and care choices; (4) The suffering of health professionals; (5) Training evaluations and future expectations. At the end of Pandemic period, new training needs and acquisition have emerged. Palliative care needs changed over time: the COVID-19 themes are now far from their perception, and somehow the skills acquired during the intensive training are less present.

**Conclusion:**

The pandemic led to a rapid acquisition of competencies and changes in the professionals’ behaviors, maintenance of professionals’ knowledge and competencies at two months and one year. COVID has improved relationships and increased interactions with the infectious world but that it has not been enough. The integration between PC and Infectious world needs models of integration to implement.

## Introduction

The role and response of palliative care services (PCS) around the world during the pandemic were exceptional, especially in the hospital setting (1–4). Moreover, in emergency departments (EDs), PCS largely contributed to physical/spiritual/social symptom control, staff support and decision-making for COVID-19 patients (5–9).

This substantial effort was realized by reorganizing PCS to better respond to the increasing patient and hospital needs (10). Flexibility and adaptability by PCS have shown key characteristics to counteract the pandemic (6, 8). Even during the pandemic, PCS exercised their advanced skills on other healthcare professionals (HPs), according to the model from the 2nd level (specialist) to the 1st level (non-specialist) professionals (11). Educational activities have the goal of improving both the quality of patient care in their specific ward and the medical education that novice professionals receive (12).

Training on basic palliative care (PC) competencies and the knowledge of other specialists was also essential to expand the health care system to provide PC; on the other hand, adapting PC competencies, preferably with short and focused teaching or tutoring, was important due the emerging new scenarios, including patients’ use of communication devices, the limited time available for the delivery of care and managing death inside the hospital and relationships with family members outside of the hospital (5, 6, 13–15).

The published literature on models of integration between palliative and infectious care is non-existent; there are several calls for CP for HIV (16–19) patients in light of the great palliative care needs but no model already implemented (20). The literature between these two worlds focuses very much on the appropriateness of prescribing antibiotics in certain special situations such as the advanced cancer patient (21) or the patient with advanced dementia (22–25) or in infectious control in PC setting (22, 23). PC is underrepresented in global guidelines for responding to infectious disease outbreaks (24). Most of the literature has flourished in the last years due to the COVID pandemic.

Our PCS is a specialized hospital-based unit with no dedicated beds in a Comprehensive Cancer Center in Northern Italy. The PCS was established in 2013, and its goals included developing and implementing training programs to improve health professionals’ PC competencies (25, 26).

In March 2020, during the first wave of the pandemic, we joined a strong collaboration with the Infectious Disease Unit, a front-line department, to implement protocols for symptom control (especially dyspnea, delirium and end of life), support staff in delivering bad news and advance care planning, and control emotional distress and communication with families through intensive training (15). Following the PCS intervention, we explored the experience of HPs in PC. Our study suggests that a PCS can help in managing symptoms and answering the new needs emerging due to the COVID-19 outbreak: both supporting staff and having an educational role in training non specialist HPs; dealing with atypical relationship of care and giving difficult news to isolated patients and to their family members; guaranteeing personalized care (15).

In this article, we focused on PC needs and their changes perceived by HPs before the pandemic and after intensive training. We qualitatively evaluated the impact of the training itself and the maintenance of professionals’ knowledge and competences at two months and one year. We used individual, semistructured interviews to get a deeper understanding of the professional’s experience, while the two validation meetings were used to confirm and/or challenge our findings, adding a group (entire ward’s staff of infectious disease) perspective to what we previously found and to assess the educational needs to support future training. Knowledge of PC needs and their changes could be an element to implement a new integration model between PC and the infectious world.

To the best of our knowledge, this is the first longitudinal qualitative study on meaning shifts in PC needs and competencies before, during and after COVID-19. We believe that this longitudinal research could provide new insight into PC delivery in emergency situations and beyond.

## Materials and methods

### Study design and participants

We developed a satellite, qualitative study to add detail to what was retrieved in a first case study published in 2020. From March 9 to 28, 2020, the PCS developed intensive experiential training with specific component: supervision of 18 daily briefings with nine Infectious Diseases Unit physicians; 48 PC bedside consultations together with the referring physician; two brief (30–40 min) lectures on PC topics, with a PC expert answering the Infectious Diseases Unit physicians’ and nurses’ questions; a booklet addressing the assessment and treatment of PC needs based on issues emerging during the first week of the intervention and elaborated for use by professionals (15). Thirty-one HPs of the Infectious Diseases Unit (physicians and nurses) participated in the training. The intervention was applied to all staff of the Infectious Diseases Unit who were facing the COVID-19 emergency. In 2020 and 2021, these HPs visited approximately 1,022 and 1,401 COVID cases, respectively. No one refused the intervention.

A longitudinal qualitative research design using semi-structured interviews followed by thematic analysis (27–29) was utilized. This specific thematic approach enabled the researchers to fully understand the experiences of the HPs after they participated in the intensive PC training program. Study procedures and reporting followed the Consolidated Criteria for Reporting Qualitative Research (CoreQ) guidelines (30).

**Figure 1:**
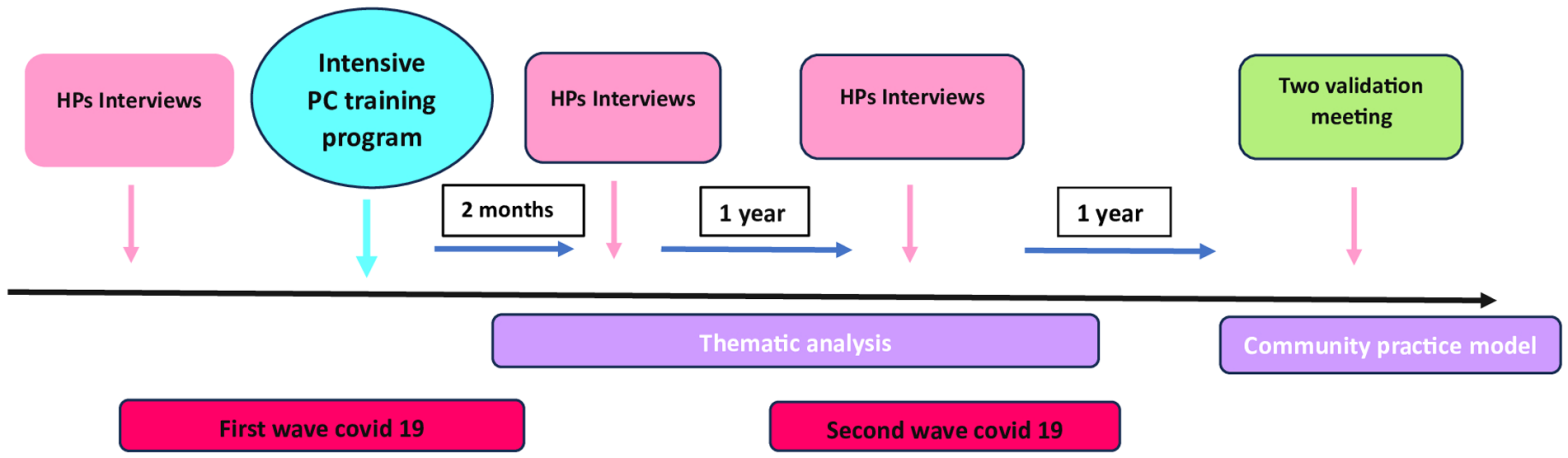
A graphic description of the whole intervention along with its evaluation.

Figure 1 describes the timeline of the whole intervention along with its evaluation.

### Data collection

Eight HPs (four physicians and four nurses), accounting for 25% of the professionals who participated in the intensive training realized in 2020 (15), were recruited by researchers drawing numbers. No one refused the invitation to participate in the interviews and no HPs were lost to follow up. Eight semi-structured interviews of the same professionals were performed during the first wave, two months (June 2020) after training (T1), and during the second wave (T2), one year (May/June 2021) after training (also referred as “participant validation”) (31). The last two interviews did not yield new data but served to confirm the analysis (data saturation).

The individual interviews were structured to follow a topic guide (the interested reader can find it in Supplementary material online) to explore the PC issues that the professionals encountered in clinical practice before the COVID-19 pandemic and during the pandemic, with a specific focus on the training that was carried out. All interviews were conducted by a researcher (GA) experienced in qualitative research methods (32). Written informed consent was obtained from all the participants. The date, time and site of the interview were agreed upon by the researcher and HPs. Explicit permission was given for the interview to be audio-recorded. The interviewer did not meet or know the participants before the study.

One year after T2, we presented the results of our analysis to the ward’s staff through two “validation meetings.” These validation meetings were focus groups opened to the entire ward’s staff, one year after T2, in order to plan new, future education projects targeting the Infectious Disease Unit.

The meetings were agreed upon with the formal leadership of the ward. We scheduled two editions of each meeting, to maximize participation.

Each meeting had two facilitators, co-authors of the previous paper, and one observer. Twenty-two HPs participated, 8 physicians and 14 nurses.

Our effort was aimed at gaining a deeper understanding of the perception of their current educational needs as a “community of practice” (as described in the “discussion” section), and not only at an individual level (see Figure 1).

### Data analysis

The results of the interviews were analyzed through thematic analysis to identify any possible changes in the meanings attributed to that phenomenon from T1 to T2 (27, 28). The same researcher who conducted the interviews conducted the verbatim transcription. On average, each interview lasted for 25 min. The interviewer did not return the transcripts to the participants. Each transcript was labeled independently by two researchers (SA; ST). Through an iterative process during the analysis, the researchers verified that, from time to time, the main sub-themes and the themes of the content that comprised them were consistent with the transcribed interviews and identified significant sentences that condensed and represented the meanings of the identified sub-themes and themes. Then, the labels were combined to identify the central themes and sub-themes. The two researchers compared their analyses and proposed a shared naming. The themes were further reviewed by G.A. to ensure accuracy. The list of identified themes was discussed and refined by the entire team of researchers to ensure internal consistency. The central themes were accurately described to identify their meanings and the change in their meanings over time (29, 32, 33).

All the identified final themes were saturated, with the last interviews not yielding new insight or new data but serving to confirm the analysis.

The validation meetings were performed in according with the best practices in medical educational (as described in Kern, Thomas and Hughes, having a collective, formative evaluation of the group perception of the previous training is helpful in planning a better future training’s edition) (34). In the meetings we presented our results and gathered their perception of their current educational needs. To do so, we asked them to describe clinical cases where they detected palliative care needs.

These data were then compared with the previous results in search for any significant shift.

This study received ethical approval from the Ethics Committee AVEN of the Azienda USL-IRCCS Reggio Emilia in May 2020 (n° 2020/0047973).

## Results

The participants’ mean age was 46 years, their time since graduation ranged from one month to 31 years, and the median years of work experience were 21 years (see Supplementary material online).

We present the results in two sections: in the first section, we describe the themes identified in the analysis of the longitudinal interviews, at T1 and T2 (see Table 1). In the second section, we describe the feedback on our analysis and the new educational needs declared by the ward’s staff 12 months after T2, at the beginning of a new educational project.

**Table 1:** Thematic analysis.

Theme/sub-theme	Before training (T1)	Two months after training (T1)	One year after training (T2)
THEME 1: Recognizing patients’ palliative care needs			
What types of PC needs?	From: The need for palliative care is linked just to sedation and difficult communications	To: Failure to identify increased palliative care needs	To: Strong perception of the isolation of patients with COVID-19.
THEME 2: Responses to palliative care needs			
What types of responses to palliative care needs?	From: Not knowing how to respond or responding with drugs or techniques	To: Multiple and complex answers	To: The competence has become the feature of the team
THEME 3. Increasing attention to intervention and care choices			
Perceptions, competence and emotions	From: Perceptions, helplessness and unsuitability	To: Development of skills in palliative care	To: The emotions became glaring
Conflicts and ethical reflections	From: Contrast of opinions for the treatment choice	To: Solutions to even complex cases are found	To: Developed reflections on ethical problems
THEME 4. The suffering of HPs			
The support of HPs	From: High level of stress for professionals	To: Reduction of emotional stress	To: Onset of fatigue for the team
THEME 5: Training and future expectations			
Short and long-term perceptions	___	From: Great satisfaction	To: the training was “archived”

We decided to present results in such form, as the trainee needs and attitudes changed significantly over time, and the group feedback meetings from the staff were upmost informative and helped us to developing a plausible explanation of the perceived, relatively low retention of the training contents.

### Interviews thematic analysis

The analysis of the interviews led us to identify five overarching themes (Table 2) (1): Recognizing patients’ PC needs (2); Responses to PC needs (3); Increasing attention to intervention and care choices (4); The suffering of HPs; and (5) Training evaluations and future expectations. These themes emerged with different meanings (defined within the subthemes) in relation to pretraining and two months and one year post-training.

**Table 2:** The main results of the interviews.

During the COVID-19 first wave’s experience, the isolation of the patient was perceived as a source of intense suffering by health professionals Palliative Care skills were perceived as a team acquisition The overall experience was highly emotional for the staff’s members The PC training led to a stronger focus on ethical considerations in everyday practice The overall educational experience was perceived as a circumscribed experience, mostly positive

#### Theme 1. Recognizing patients’ palliative care needs

In this theme, we denominated the meaning shift changing from *“The need for palliative care is linked just to sedation and difficult communications”* to *“failure to identify increased palliative care needs”* two months after training, to *“strong perception of the isolation of patients with COVID-19”* one year after training. We identified a subtheme denominated “What types of PC needs?.” Before training, the HPs identified various types of palliative care needs and the need to receive basic relevant information on PC. For example, the HPs identified palliative sedation, difficulty communicating with patients and their families, symptoms that are difficult to treat, and managing patients’ psychological problems.

*“The difficulty communicating with patients was very lacking…” (T1 Cod. 7.24-Physician)*

“*They needed more intensive care and there was a need to set up a sedative therapy quickly…” (T1 Cod. 1.14—Physician).*

After training, the HPs identified the isolation of patients as PC need:

*“…one problem is patient isolation…has not been resolved and no major interventions have been made” (T2 Cod. 2.23—Nurse).*

#### Theme 2. Responses to palliative care needs

In this theme, we identified a sub-themes called: “What types of responses to PC needs?.” As a result of the training, the meanings shifted from what we denominated *“Not Knowing how to respond or responding with drug or techniques”* to *“multiple and complex answers”* to *“the competence has become the feature of the team.”* Before the training, the HPs primarily emphasized the lack of a specific approach to PC needs. They recognized responding with only pharmacological interventions or technical interventions for acute symptoms. They often employed psychologists to address other dimensions of quality of life.

*“more than with elastomer or long-term therapies, we dispensed medications as needed…” (T1 Cod. 1.18—Physician)*

*“…empirically, we talk to family members, we try to understand if they are aware” (T1 Cod. 7.24—Physician).*

After training, the HPs recognized that they responded more effectively and comprehensively to PC needs:

*“…we have improved dyspnea management…pain management…generalized pain related to the lack…you pass me the term…of life” (T1 Cod. 3.24—Nurse)*;

*“…we have acquired expertise in the management of delirium and agitation” (T1 Cod. 5.4—Nurse).*

One year after training, the acquired skills were assets for the team, and the situation was more manageable: *“we were already tested, organized and we knew what it was all about…” (T2 Cod. 8.17—Nurse)*. Nevertheless, the problem of identifying psychosocial and spiritual needs remained in both the pre- and post-training interviews.

#### Theme 3. Increasing attention to intervention and care choices

In this theme, two sub-themes were identified (1): perceptions, competence and emotions and (2) conflicts and ethical reflections.

##### Perceptions, competence and emotions

The participants shifted from what we called *“Perceptions, helplessness and unsuitability”* to the *“Development of skills in palliative care”* to *“the emotions become glaring.”* Before training, the HPs perceived a great sense of inadequacy and discomfort, mainly due to their lack of knowledge of the new situation. Furthermore, they realized that their interventions were not very effective.

*“It was considerably uneasy for us as professionals because we could not…how to say…to manage the…the pain of that moment because it was completely new for us” (T1 Cod 3.20—Nurse).*

After training, the HPs recognize that they have developed skills in symptom management and acquired the importance of multidisciplinary work.

*“…the experience of COVID-19 has taught that this pathology must be managed in a multidisciplinary way” (T1 Cod 7.20—Physician).*

The HPs lead the patients’ pathways with other departments and developed *“awareness that older adult patients, with dementia, die with dignity.” (T1 Cod. 7.13—Physician).*

After one year, despite the skills acquired, the emotions of the HPs become evident for *“not seeing a way out soon” (T2 Cod. 1.24—Physician)*.

The HPs are tired of always managing and only managing this pathology and *“…often you felt helpless because you could not find any positive feedback.” (T2 Cod. 8.30—Nurse).*

##### Conflicts and ethical reflections

The participants shifted from what we called *“Contrast of opinions for the treatment choice”* to *“Solutions to even complex cases are found”* to *“Developed reflections on ethical problems.”* Before the training, palliative sedation was a cause of conflict among the nurses and physicians. The HPs had difficulty managing ventilated patients and patients who asked to be sedated.

*“…doctor, this patient is too agitated and if we put C-PAP, she will not tolerate it! The doctor replied: ‘Let us try without sedation’. After a few hours, the patient had torn C-PAP and bladder catheter…” (T2 Cod. 2.20—Nurse)*

*“I remember a patient…who said: ‘I absolutely do not want to feel difficulty of breathing, if it goes badly, fall asleep, fall asleep’” (T1 Cod. 1.16—Physician).*

After training, the HPs described the capacity to manage complex cases and greater knowledge of the indications for palliative sedation. The participants discovered the importance of end-of-life (EoL) treatments, as higher sensitivity and attention were given to patients.

*“An old woman began to breathe poorly…she was beginning to be delirious…making phone calls her daughter saying ‘They keep me prisoner’…we used elastomer and she said ‘I’m better, I breathe better.’ She died, but she died quietly” (T1 Cod. 1.27—Physician).*

Over time, after training, the HPs begin to ask ethical questions, and they were more critical of decision-making. The HPs began to investigate ethical issues such as the need for some invasive treatment, respect for the dignity of the person and not ventilating patients. The HPs claimed:

*“[In the second wave] the situation seemed much clearer to us. In the first wave age was a dramatic factor and there were many discussions…” (T2 Cod. 1.12—Physician)*

*“We have discussed several times about the use of the nasogastric tube during ventilation… also for an invasiveness and dignity of the body” (T2 Cod. 2.16–17—Nurse).*

#### Theme 4. The suffering of HPs

Another theme emerging from interviews regarded the difficulty of the HPs in sustaining, over time, the emotional charge of daily contact with patients who have COVID-19 and their families. The sub-theme is represented by the support to HPs. The participants shifted from what we called *“High level of stress for professionals”* to *“Reduction of emotional stress”* to *“Onset of fatigue for the team.”*

Before the training, there was evident suffering on the part of the professionals, especially nurses, due to the reduced use of the appropriate drugs for sedation.

*“I suffered a lot because if you have the opportunity, I did not understand why not being able to use them [the drugs] and not being able to give the patient less suffering” (T1 Cod. 3.19—Nurse).*

Following training, the HPs recognized, above all, the need to receive psychological support themselves to face complex COVID-19 and EoL situations. The HPs recognized the training as an occasion for discussion and sharing of their most complex problems: *“In my opinion, the shared decision-making helped me a lot, it personally helped me a lot.” (T1 Cod. 1.22—Physician).*

Furthermore, the help of the PCS in reducing the patients’ suffering “…*reduced the medical and nursing staff’s suffering who were the ones most in contact… (T1 Cod. 1.25—Physician).*

Over time, the HPs’ fatigue developed. The participants were tired of managing only this disease: *“It lasted little over two months and then we started again…we are worn out by a situation that has destabilized us.” (T2 Cod. 8.10–20—Nurse); “It’s quite frustrating, to see new cases again…” (T2 Cod. 1.24—Physician).*

#### Theme 5. Training and future expectations

In this theme, the sub-theme identified has been called “short and long-term perceptions.” The participants shifted from what we called *“Great satisfaction”* to *“the training was ‘archived’” (T2 Cod. 4.16—Physician).* The HPs recognized that they have acquired some skills to deal with the needs of PC, and they appreciated the method based on clinical practice. The collaboration with PCS has brought greater well-being for patients and also HPs.

*“For me it was useful…it dissolved my doubts about sedation, being unable to understand why the physicians are so reluctant, I had confirmation…” (T1 Cod. 2.30—Nurse)*

*“…this is new, very, very useful…” (T1 Cod. 3.33—Nurse) “A huge thank you” (T1 Cod. 1.35—Physician).*

The HPs’ perceptions were that the second wave of COVID-19 *“…canceled everything that was done before because getting back into the COVID routine was difficult” (T2 Cod. 4.15—Physician)* and *“In my opinion it was archived” (T2 Cod. 4.16—Physician).* After one year of training, they feel the need for continuous training that could promote a change in their mentality with respect to palliative medicine.

*“We abandoned it too quickly…From too much to zero; there was a challenging gap” (T2 Cod. 7.33—Physician)*

*“…for some situations every now and then, some refresh is useful…” (T2 Cod. 1.10—Physician).*

### Ward’s staff group feedback at 12 months from T2

We inquired the ward’s staff feedback in two validation meetings: in the first meeting we presented the results of previous interviews and study (15) (Table 2) and asked for their current perception of these results and how much they might have changed over time.

In the second meeting we asked them to define Palliative Care from their perspective and to present real life situations and cases where they faced palliative care needs as a ward (Table 3).

**Table 3:** Example of real case with PC needs as perceived by HPs.

Case
An in-patient, amputee, dysphagic, dialysed, cognitively impaired, mutilated patient.
Caregivers who insist on feeding him by oral
Frequent and long admissions (last 1 month) for recurrent ab ingestis sepsis
**Needs identified by the group:**
Social: ineffective caregiver
Bioethics: appropriate PEG placement? overtreatment in antibiotic treatment?
Different views of careers
Lack of awareness of the stage of the disease on the part of the career
Medical-legal complexity
Discontinuity with community services

When asked to define their perception of palliative care needs, they focused mainly on treatment of pain (nine staff members), and psychological end of life needs (four staff members), and most of the participants referred to palliative care as EoL care (12 of them).

Table 4 summarizes the perceived current needs at T2 + 12 months, (compared to Table 2, reporting the needs at T1 and T2).

**Table 4:** Ward’s staff group feedback at T2 + 12 months main perceived needs.

Patient isolation is no longer perceived as problematic in everyday work New needs emerge: How to treat in a more human and competent way the patients affected by dementia, delirium, or other conditions often leading to physical restraint? Questions about treatment suspension and current law’s indications (recently changed in Italy) Symptom management (pain and sedation) Questions on how to better manage CALD (culturally and linguistically different) patients, especially regarding religion and death How to break bad news, especially to patients with oncologic and hematologic disease Observers noticed that the perceived higher closeness to patients seems gone, and a renewed, stronger asymmetry in the relationship emerges (stronger use of technical terms and a technical approach when dealing with highly activating situations) Questions on appropriateness of PC consultations in care proportion discussions (ICU referral/EoL care?)

As you can see, palliative care needs changed back significantly over time: the COVID-19 themes are now far from their perception, and somehow the skills acquired during the intensive training are less present.

## Discussion

Our findings describe the experience of the HPs of the Infectious Diseases Unit after intensive training in PC; the study explored the participants’ learning needs prior to the pandemic, during the first wave and one year later. We explored their perceptions, in terms of educational needs and feedback 12 months after T2, at the beginning of a new educational project (Table 4), and their palliative care perception was in part back to a EoL care and symptoms management perception.

Few studies have reported whether educational interventions have longitudinal impacts on behaviors and results (35). A longitudinal evaluation was useful, for example, to underline PC competences.

Although PCS has implemented a short training, HPs expressed great satisfaction with the method based on clinical practice with quick responses. The HPs recognized the training as an occasion for the discussion and sharing of the most complex problems. The shared search for common solutions became a support strategy, and the help of the PCS unit in reducing the patients’ suffering also led to a reduction in the staff’s suffering (36).

As suggested by other authors (37), the presence of a mentor meant that these questions and insights could be explored and clarified quickly, thus leading to a faster learning process (38, 39).

Once again, this confirms the supporting role of the PCS that stimulates reflection on complex cases, but also an important role in simultaneous care.

In support of this important role, it should be emphasized that one year after the training, it emerged that the professionals have seen the training archived too quickly. Having lost the continuous and lasting comparison over time has brought out glaring emotions and fatigue for the team. Some physicians’ competences seem to decrease, and some clinical confidences increase (40).

Moreover, as in other published experiences (26, 41, 42), the participants experienced an impact of the team training program on interprofessional collaboration in the ward and suggested that team-based learning supports the transferability of knowledge to clinical practice and the need for continuing CP training.

The main changes analyzed in this study such as patient isolation, staff support, maintenance of basic CP skills and ability to recognize complexity and ethical issues are the subject of a new training program for HPs, initiated by the two meetings at T2 + 12 months. It’s important to notice that this perception seems to be somehow lost over time, as well as training effect.

While many explanations are possible, we’d like to highlight some considerations in terms of “community of practice.” J. Lave and E. Wenger describe learning as a mainly social process, “situated” in the participation of a social context. *A community of practice is “group of people who “share a concern or a passion for something they do and learn how to do it better as they interact regularly,” and have some structural characteristics* (see Table 5) (43).

**Table 5:** Community of practice.

Community of practice’s characteristics according to Wenger	Our experience
Domain: A domain of knowledge establishes common ground, motivates members to participate, directs their learning and gives their actions significance.	COVID-19 symptom management and emergency (Now the common ground is mainly represented by symptom management in patients with oncologic disease)
Community: The concept of a community provides the social framework for this learning. Strong communities encourage interaction and the willingness to share ideas.	Participation of PC staff to ward’s activity and meetings (now the interaction is limited to referral, mainly in case of end-of-life care)
Practice: While the domain provides the general area of interest for the community, the practice is the focus around which the community develops, shares and maintains its knowledge base.	Bedside consultations, phone call breaking-bad-news sessions, meetings and briefings where highly relevant, sometimes life-or-death decisions where shared (a shared practice between the two groups is now not clearly detectable)

We believe that the intense COVID-19 experience led to the provisional creation of a real “community of practitioners,” where PC professionals were perceived as part of the same social group as the infectious disease ward’s staff.

We can find many possible explanations for why this phenomenon faded, at least partially. According to Wenger’s theorization, one possible explanation might be: the identity of the infectious disease ward’s staff dramatically changed, but is now back to an “older version,” as COVID-19 has a significantly smaller social impact. In 2020, when they were living the new identity of professionals facing an unprecedented social danger, this new identity facilitated the creation of a new community of practice.

While the relationship between the two Units is still good and well-functioning, it’s not possible to distinguish the same characteristics of a shared community of practitioners (see Table 5).

Probably, the local, temporary change of identity did not match an equivalent change of identity at a more global level (i.e., the infectious disease staff worldwide did not change their attitude toward PC as a group) and the specific needs of that time catalyzed a quick change, that wasn’t sustained by the same needs over time. From 2020 to 2022, the initial consultations carried out by the PCS have decreased. Especially way, in 2020, the PCS performed 63 initial consultations, while in 2022 performed 19 initial consultations.

Our experience shows that COVID has improved relationships and increased interactions with the infectious world but that it has not been enough; as in all educational experiences, continuous supervision of needs, monitoring and evaluation over time is necessary. The needs of patients admitted to these wards are multiple and infectious specialist clinicians perceived themselves unable to respond to them. Moreover, the data indicated that PC education may need to be tailored to meet the needs of different medical specialties (42) so all HPs can provide primary palliative care (6).

Our model, consisting of a cycle of assessment of educational needs and training, suggested an effective way to assess the real retention of interest, skills and knowledge in PC in an infectious disease ward after COVID-19. In such an innovative and uncertain field, the iterative evaluation of educational needs might effectively support the training of HPs, and help PCS maintain flexibility and adaptability mentoring approach without taking for granted the acquisition of skills. Furthermore, analyzing how the needs of the ward’s staff change over time will allow the PCS to reorganize itself to create the conditions for more effective and long-lasting collaborations with infectious disease word.

## Strengths and limitations

As typical in qualitative research, these data can help researchers and decision-makers generate hypotheses for those who face similar challenges but are not strongly generalisable in contexts different from ours.

Our study described an experience of a single institute. The number of interviews was small but sufficient to have a saturation of the results and not have new themes to explore. Another limitation of our study was the potential recall bias and possible selection bias in the participants. However, at the same time, the longitudinal characteristic and the analysis with different methodologies may have reduced these limits.

The results provide an in-depth understanding of HPs’ experiences with the implementation of a longitudinal team training program that may also offer relevant knowledge for other similar health-care settings.

## Conclusion

Palliative care is a complex and difficult discipline to be learned. The pandemic led to a rapid acquisition of competences and changes in the professionals’ behaviors.

PC professionals should maintain a mentoring approach over time and tailor training to the changing needs of HPs.

Specific models of integration between palliative care and infectious diseases are needed, to be tailored to the specific needs of these patients, which are not limited to COVID Pandemic moments.

## Data Availability

The datasets presented in this study can be found in online repositories. The names of the repository/repositories and accession number(s) can be found in the article/Supplementary material.
