# Supplementary material for: Training an infectious disease unit in palliative care during and post COVID-19: a qualitative longitudinal study
Source: Front Public Health. 2024 Oct 16;12:1393770. doi: 10.3389/fpubh.2024.1393770 (PMC11521919; doi:10.3389/fpubh.2024.1393770)
Supplement: Supplementary file 1 [file Table_1.docx]

**HP’s interview guide**

| **T1. First wave/two months after intensive training**  Theme 1 *PC needs before the COVID-19 pandemic:*  The interviewer investigates the identified PC needs and the clinical behaviours of HPs in dealing with complex issues before the COVID-19 pandemic and during the pandemic.  Sample questions: “What problems or PC needs existed before the COVID-19 emergency? What behaviors did you adopt to deal with complex clinical problems before the COVID-19 emergency?  Theme 2 *The PC needs highlighted by the COVID-19 emergency:*  The interviewer investigates the methods used to identify PC needs and problems encountered in dealing with PC needs and investigates the impact of joint interventions between clinicians and the PCS in clinical practice.  Sample questions: What problems concerning palliative treatment did you encounter during the COVID-19 emergency? What solutions are adopted before involving PCS and after their involvement? In your opinion, what results can be achieved through joint interventions with PCS?  Theme 3 *Evaluation training and the need for the consolidation of training:*  The interviewer analyzed the intensive training experience during the COVID-19 pandemic and which skills must be integrated to achieve general competence in palliative care.  Sample questions: What was your experience with the intensive training? What was your opinion on the intensive training? Do you think you have acquired skills in PC? How would you like to consolidate the training in the future? What would you like to integrate the training that was already carried out?  **T2. Second wave - one year after training**  Theme 1 *The palliative care needs highlighted by the second wave of the COVID-19 emergency*  Sample questions: What palliative care needs did you identify in the second wave of the pandemic? What solutions have been adopted after PC training?  Theme 2 *Evaluation training and the need for the consolidation of training*  Sample questions: After one year, what is your experience and/or opinion with the intensive training? Do you think you have acquired skills in PC? How would you like to consolidate the training in the future? What would you like to integrate with the training that was already carried out? |
| --- |
