# Supplementary material for: Training an infectious disease unit in palliative care during and post COVID-19: a qualitative longitudinal study
Source: Front Public Health. 2024 Oct 16;12:1393770. doi: 10.3389/fpubh.2024.1393770 (PMC11521919; doi:10.3389/fpubh.2024.1393770)
Supplement: Supplementary file 2 [file Table_2.docx]

**Participant characteristics**

| **Profession** | **Age** | **Gender** | **Work experience** |
| --- | --- | --- | --- |
| Physician | 33 | F | 3 years |
| Nurse | 48 | F | 15 years |
| Nurse | 44 | F | 6 years |
| Physician | 32 | M | 1 months |
| Nurse | 50 | F | 30 years |
| Nurse | 51 | F | 30 years |
| Physician | 59 | M | 31 years |
| Physician | 56 | F | 27 years |
